# Janus Kinase Inhibitor Discontinuation at a UK Tertiary Centre: A Retrospective Study

**DOI:** 10.7759/cureus.98018

**Published:** 2025-11-28

**Authors:** Katie Bland, Ameen Jubber, Sujata Ganguly, Arumugam Moorthy

**Affiliations:** 1 Medicine, University Hospitals of Leicester NHS Trust, Leicester, GBR; 2 Rheumatology, University Hospitals of Leicester NHS Trust, Leicester, GBR; 3 Rheumatology, The Tamil Nadu Dr. M.G.R. Medical University, Chennai, IND; 4 Rheumatology, College of Life Sciences, University of Leicester, Leicester, GBR

**Keywords:** baricitinib, filgotinib, jak inhibitor, janus kinase inhibitor, janus kinase (jak), rheumatoid arthritis, rheumatology, tofacitinib, upadacitinib

## Abstract

Objective: The aim of this study is to evaluate Janus kinase (JAK) inhibitor discontinuation rates and reasons for discontinuation in patients with inflammatory arthritis at a UK tertiary centre.

Methods: Patients prescribed JAK inhibitors (tofacitinib, baricitinib, upadacitinib, and filgotinib) from May 2018 to October 2023 were included in this study, and those who discontinued their JAK inhibitor were identified. Statistical tests were conducted to assess for associations between discontinuation reasons and patient baseline characteristics.

Results: Of 400 patients, 102 (25.5%) discontinued their JAK inhibitors. Primary inefficacy was the most common discontinuation reason, reported in 35/102 patients (34.3%), followed by side effects in 25/102 patients (24.5%). Discontinuation rates varied among the drugs, with tofacitinib (33/64; 51.6%) and baricitinib (57/243; 23.5%) having higher overall discontinuation rates, and upadacitinib and filgotinib showing higher early discontinuation.

Conclusion: JAK inhibitor discontinuation is primarily driven by inefficacy and side effects, with variations by drug. Further studies are needed to explore long-term safety.

## Introduction

Janus kinase (JAK) inhibitors are an important therapeutic option in treating patients with inflammatory arthropathy, presenting an oral alternative to biologic disease-modifying antirheumatic drugs (bDMARDs) [1]. JAK inhibitors, including baricitinib, tofacitinib, upadacitinib, and filgotinib, work by inhibiting the JAK-STAT pathway, an intracellular signalling mechanism that plays an important role in the pathophysiology of many inflammatory arthropathies [2]. JAK inhibitors are efficacious in clinical trials [3-5], and there is a growing real-world literature on JAK inhibitor discontinuation [6]; however, comparative, reason-specific analyses remain limited.

Many factors can lead to discontinuation of therapy in patients with inflammatory arthropathy, including inefficacy, safety concerns, side effects, and patient preference. In recent years, concerns have been raised about the safety of JAK inhibitors, especially following the ORAL (Oral Rheumatoid Arthritis Trial Programme) surveillance study, which identified an increased risk of cardiovascular events and malignancy in patients receiving tofacitinib compared to tumour necrosis factor inhibitors (TNFi) [7]. This was reflected in the 2022 European Alliance of Associations for Rheumatology (EULAR) recommendations for the management of rheumatoid arthritis (RA) [8] and has also led to safety warnings from regulatory bodies, such as the Medicines and Healthcare products Regulatory Agency (MHRA) [9] in the United Kingdom (UK), that may influence prescribing behaviour and lead to higher discontinuation rates.

The objective of this study was to evaluate JAK inhibitor discontinuation rates and the reasons for discontinuation among patients with inflammatory arthritis treated at a UK tertiary centre. Specifically, we aimed to compare discontinuation rates across individual JAK inhibitors (tofacitinib, baricitinib, upadacitinib, and filgotinib), categorise and analyse the reasons for discontinuation, and explore associations between these reasons and baseline clinical characteristics, including age, ethnicity, disease activity, and concomitant treatments. We hypothesised that discontinuation patterns would vary between different JAK inhibitors and that certain patient characteristics may influence the reasons for discontinuation. 

## Materials and methods

Study design and patients 

**Figure 1:**
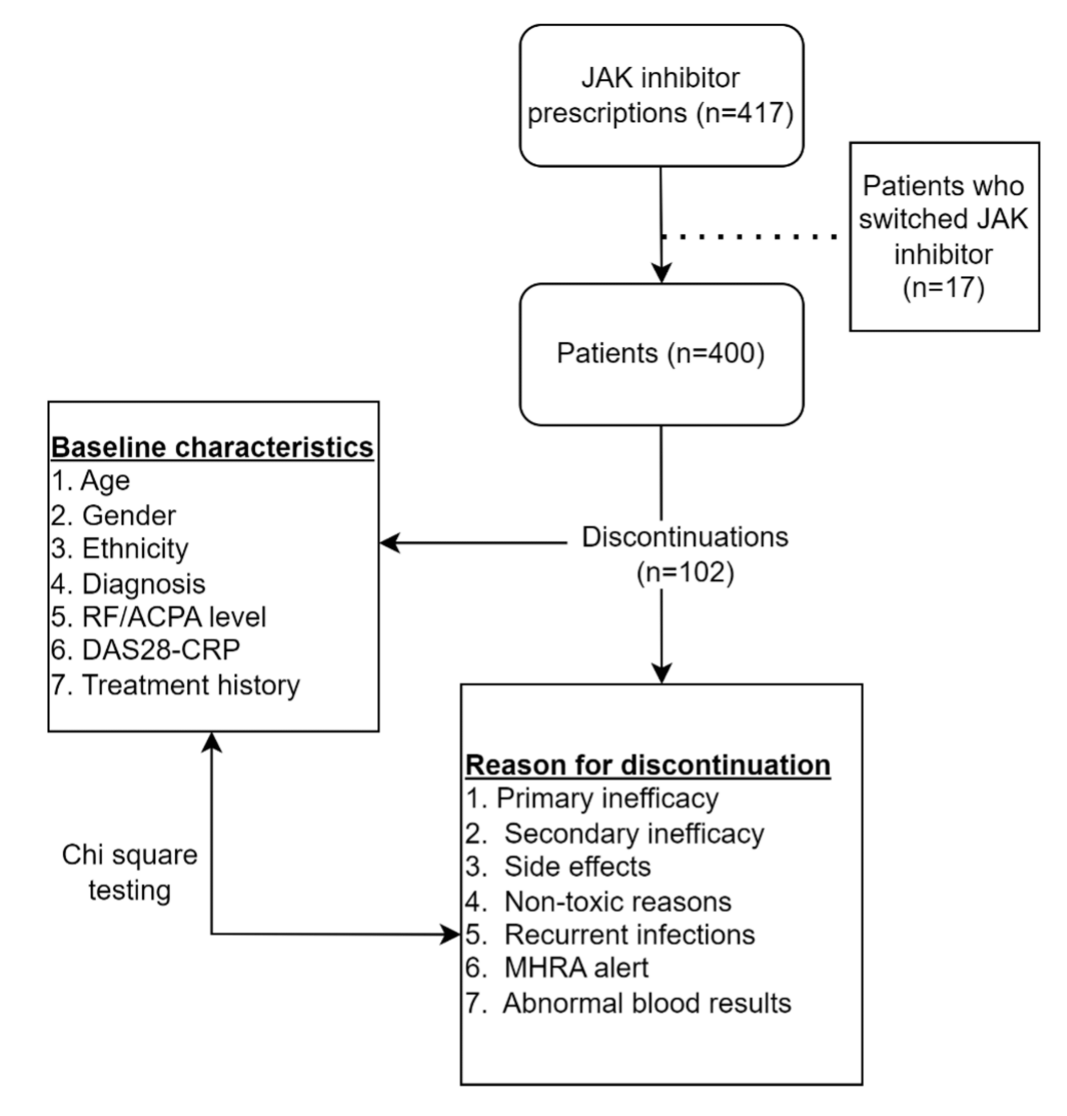
Flow diagram of patient identification, baseline assessment, and discontinuation analysis JAK: Janus kinase; RF: rheumatoid factor; ACPA: anti-citrullinated protein antibody; DAS-28 CRP: Disease Activity Score in 28 joints using C-reactive protein; MHRA: Medicines and Healthcare products Regulatory Agency

This was a retrospective, single-centre study carried out at the University Hospitals of Leicester, Leicester, UK. All JAK inhibitor prescriptions between May 2018 and October 2023 were identified from an electronic pharmacy and biologic monitoring database used by the rheumatology service. All patients who had been prescribed one or more JAK inhibitors (tofacitinib, baricitinib, upadacitinib, or filgotinib) for rheumatological indications were included in the study. Patients were identified using prescription records cross-referenced with electronic clinic letters to confirm the diagnosis and indication for treatment. The patient selection process is shown in Figure 1. 

Exclusion criteria included: (1) patients prescribed a JAK inhibitor outside the rheumatology service (e.g., dermatology or gastroenterology indications); (2) patients with incomplete medical records such that discontinuation status or reason could not be determined. No patients were excluded based on age, gender, ethnicity, or comorbidities. 

Data collection 

Each patient’s electronic medical records were used to determine the type of JAK inhibitor they were prescribed, whether it had been discontinued, and the start and stop dates. Data were extracted manually using a standardised proforma to ensure consistency. All data were anonymised before analysis, stored on secure NHS servers, and accessible only to authorised study investigators. Baseline characteristics were also recorded, including age, gender, diagnosis, rheumatoid factor (RF) and anti-citrullinated protein antibody (ACPA) levels in the seropositive RA patients, and baseline Disease Activity Score-28 (DAS28-CRP) in the RA patients. We also recorded whether each patient was on concomitant glucocorticoids or a conventional synthetic DMARD (csDMARD) and their history of previous biologic or JAK inhibitor therapy. 

Disease activity was assessed using the DAS28-CRP instrument [10-14]. DAS28-CRP scores were calculated using the following components: 28 tender joint count, 28 swollen joint count, patient global assessment (Visual Analogue Scale (VAS)), and C-reactive protein (mg/L). The final score was computed using the validated DAS28-CRP formula. Interpretation was as follows: remission (<2.6), low disease activity (2.6-3.2), moderate disease activity (3.2-5.1), and high disease activity (>5.1).

For those who discontinued their JAK inhibitor, reasons for discontinuation were categorised into seven groups: 1) primary inefficacy; 2) secondary inefficacy; 3) side effects; 4) non-toxic reasons; 5) recurrent infections; 6) MHRA alert; 7) abnormal blood results. 

'Non-toxic reason' refers to cases where the drug was discontinued due to disease remission or patients planning pregnancy. The MHRA alert refers to cases where the pharmacy team highlighted patient risk factors (given ORAL surveillance data and the black box warning) to the clinical team, leading to a joint patient and clinician decision to discontinue their drug. 

Statistical analysis 

Continuous variables such as age, RF and ACPA levels, and DAS28-CRP were expressed as mean ± standard deviation. The number of previous biologics and the number of concomitant DMARDs were expressed as median ± interquartile range. SPSS was used for statistical analysis. The chi-square test was used to compare categorical variables, and continuous variables were compared using one-way ANOVA, and when the assumptions were not met, the Kruskal-Wallis test was used. A two-tailed p-value < 0.05 was considered statistically significant. 

## Results

Baseline characteristics 

In total, 400 patients had been prescribed one or more JAK inhibitors between May 2018 and October 2023. There were 417 JAK inhibitor prescriptions since 17 patients were switched from one JAK inhibitor to another. There were no patients who had more than two JAK inhibitors. During this time, 102 patients discontinued their JAK inhibitors. Included in this group were patients who discontinued one JAK inhibitor and started another. The proportion of discontinuations for each drug was as follows: baricitinib, 57/243 (23.5%); tofacitinib, 33/64 (51.6%); upadacitinib, 6/61 (9.8%); and filgotinib, 6/49 (12.2%). The baseline characteristics of the patients are shown in Table 1. The mean age at the time of JAK inhibitor initiation was 58.1 ± 13.6 years, and most patients were female (87.3%). All patients had a diagnosis of either seropositive RA, seronegative RA, or psoriatic arthritis (PsA). In the seropositive RA patients, mean ACPA and RF levels were 154.6 ± 149 U/ml and 147.2 ± 262.6 IU/ml, respectively. In the RA patients, the mean DAS28-CRP before JAK inhibitor initiation was 5.3 ± 1. 

**Table 1:** Baseline characteristics of the patients Values are presented as mean ± standard deviation (SD) for continuous variables, and N (%) for categorical variables. RA: rheumatoid arthritis; RF: rheumatoid factor; ACPA: anti-citrullinated protein antibody; DAS28-CRP: DAS28-CRP: Disease Activity Score in 28 joints using C-reactive protein

Baseline characteristics	Tofacitinib (n = 33)	Baricitinib (n = 57)	Upadacitinib (n = 6)	Filgotinib (n = 6)	Total (n = 102)
Age at JAK inhibitor start (years)	56.5 ± 13.1	58.2 ± 13.0	59.8 ± 19.9	63.8 ± 16.8	58.1 ± 13.6
Female patients	26 (78.8%)	52 (91.2%)	5 (83.3%)	6 (100%)	89 (87.3%)
Male patients	7 (21.2%)	5 (8.8%)	1 (16.7%)	0 (0%)	13 (12.7%)
Diagnosis					
Seropositive RA	15 (45.5%)	42 (73.7%)	3 (50.0%)	3 (50.0%)	63 (61.8%)
RF level (IU/ml)	174.5 ± 332.1	147.1 ± 254.3	108.5 ± 26.2	65.8 ± 73.9	147.2 ± 262.6
ACPA level (U/ml)	126.6 ± 135.5	154.3 ± 152.7	154.0 ± 161.1	255.0 ± 170.0	154.6 ± 149.0
Seronegative RA	7 (21.2%)	15 (26.3%)	1 (16.7%)	3 (50.0%)	26 (25.5%)
Psoriatic arthritis	11 (33.3%)	0 (0%)	2 (33.3%)	0 (0%)	13 (12.7%)
DAS28-CRP (RA patients)	5.7 ± 0.8	5.4 ± 1.0	4.7 ± 1.1	5.1 ± 1.3	5.3 ± 1.0

Concomitant and past treatments are shown in Table 2. The most common concomitant csDMARD was methotrexate (47.1%). Only 26.5% of patients were JAK inhibitor or biologic naïve, and the most used biologic before initiating a JAK inhibitor was a TNFi (63.7%).

**Table 2:** Treatment features Values are presented as N (%). JAK: Janus kinase; TNFi: tumour necrosis factor inhibitor; IL-6R: interleukin-6 receptor inhibitor; CTLA4-Ig: cytotoxic T lymphocyte-associated antigen-4-Ig; IL-17: interleukin-17; IL-12/23: interleukin-12/23

Baseline characteristics	Tofacitinib (n = 33)	Baricitinib (n = 57)	Upadacitinib (n = 6)	Filgotinib (n = 6)	Total (n = 102)
Concomitant glucocorticoid	5 (15.2%)	17 (29.8%)	3 (50.0%)	3 (50.0%)	28 (27.5%)
Concomitant methotrexate	18 (54.5%)	25 (43.9%)	2 (33.3%)	3 (50.0%)	48 (47.1%)
Concomitant leflunomide	5 (15.2%)	4 (7.0%)	0 (0%)	0 (0%)	9 (8.8%)
Concomitant hydroxychloroquine	7 (21.2%)	11 (19.3%)	1 (16.7%)	2 (33.3%)	21 (20.6%)
Concomitant sulfasalazine	8 (24.2%)	5 (8.8%)	0 (0%)	0 (0%)	13 (12.7%)
Biologic/JAK inhibitor naïve	4 (12.1%)	20 (35.1%)	0 (0%)	3 (50.0%)	27 (26.5%)
Second-line therapy	12 (36.4%)	20 (35.1%)	4 (66.7%)	2 (33.3%)	38 (37.3%)
Third-line therapy	9 (27.3%)	8 (14.0%)	1 (16.7%)	0 (0%)	18 (17.6%)
Fourth-line or beyond therapy	8 (24.2%)	9 (15.8%)	1 (16.7%)	1 (16.7%)	19 (18.6%)
Previous TNFi	26 (78.8%)	32 (56.1%)	5 (83.3%)	2 (33.3%)	65 (63.7%)
Previous IL-6R inhibitor	3 (9.1%)	11 (19.3%)	0 (0%)	1 (16.7%)	15 (14.7%)
Previous CTLA4-Ig therapy	2 (6.1%)	3 (5.3%)	0 (0%)	0 (0%)	5 (4.9%)
Previous rituximab	7 (21.2%)	19 (33.3%)	0 (0%)	0 (0%)	26 (25.5%)
Previous JAK inhibitor	4 (12.1%)	0 (0%)	2 (33.3%)	1 (16.7%)	7 (6.9%)
Previous IL-17 inhibitor	5 (15.2%)	1 (1.8%)	2 (33.3%)	0 (0%)	8 (7.8%)
Previous IL-12/23 inhibitor	1 (3.0%)	0 (0%)	1 (16.7%)	0 (0%)	2 (2.0%)

Time to discontinuation 

The proportion of drug discontinuations by three, six, 12, and 24 months is shown in Table 3. The highest proportions by three months were for upadacitinib (66.7%) and filgotinib (83.3%), and 100% of the discontinuations for these two drugs occurred by 12 months of drug initiation. However, sample sizes were smaller for these two drugs than for baricitinib and tofacitinib. Mean time to discontinuation was highest for tofacitinib (16.1±10.2 months), followed by baricitinib (9.9±9.2 months, p<0.001). 

**Table 3:** Proportion and timing of drug discontinuations Values are presented as N (%). Proportions of discontinuations between drugs at each time point were compared using chi-square tests. Mean time to discontinuation was compared using the Kruskal–Wallis test. χ²:  chi-square test; KW: Kruskal–Wallis test; df: degrees of freedom

Drug	≤3 months	≤6 months	≤12 months	≤24 months	Time to discontinuation (mean ± SD, months)
Baricitinib (n=57)	14 (24.6%)	26 (45.6%)	39 (68.4%)	53 (93.0%)	9.9 ± 9.2
Tofacitinib (n=33)	2 (6.1%)	5 (15.2%)	15 (45.5%)	27 (81.8%)	16.1 ± 10.2
Upadacitinib (n=6)	4 (66.7%)	5 (83.3%)	6 (100%)	–	3.0 ± 3.9
Filgotinib (n=6)	5 (83.3%)	5 (83.3%)	6 (100%)	–	3.0 ± 3.1
Test statistic	χ² = 23.055 (df=3)	χ² = 18.596 (df=3)	χ² = 12.245 (df=3)	χ² = 4.424 (df=3)	KW = 24.634 (df=3)
p-value	<0.001	<0.001	0.007	0.219	<0.001

**Figure 2:**
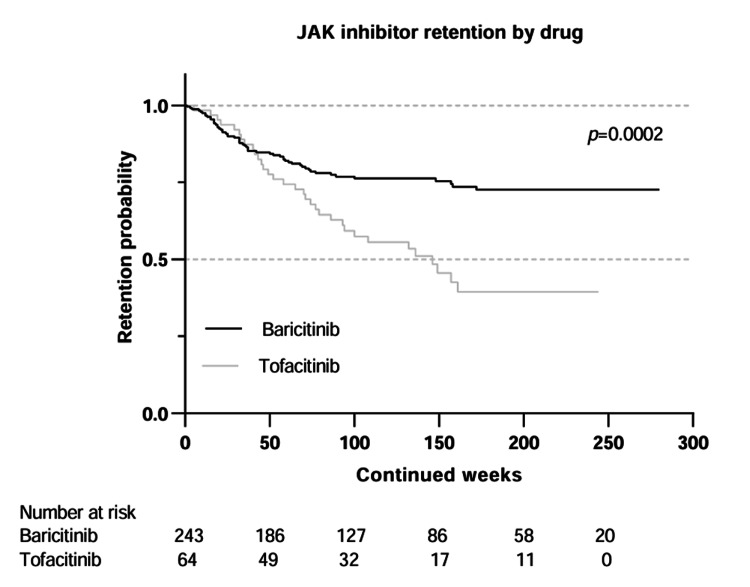
Kaplan–Meier curves comparing drug retention for baricitinib and tofacitinib Statistical comparison was performed using the log-rank test. JAK: Janus kinase

**Figure 3:**
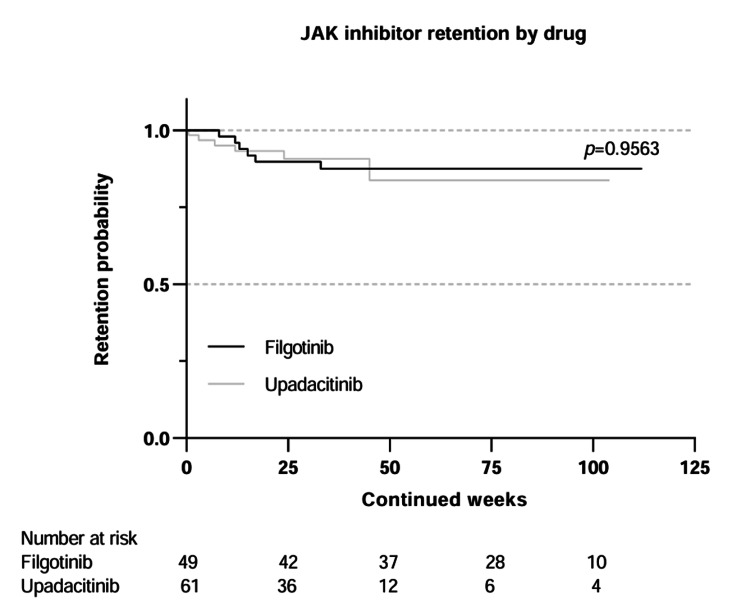
Kaplan–Meier curves comparing drug retention for filgotinib and upadacitinib Statistical comparison was performed using the log-rank test. JAK: Janus kinase

**Figure 4:**
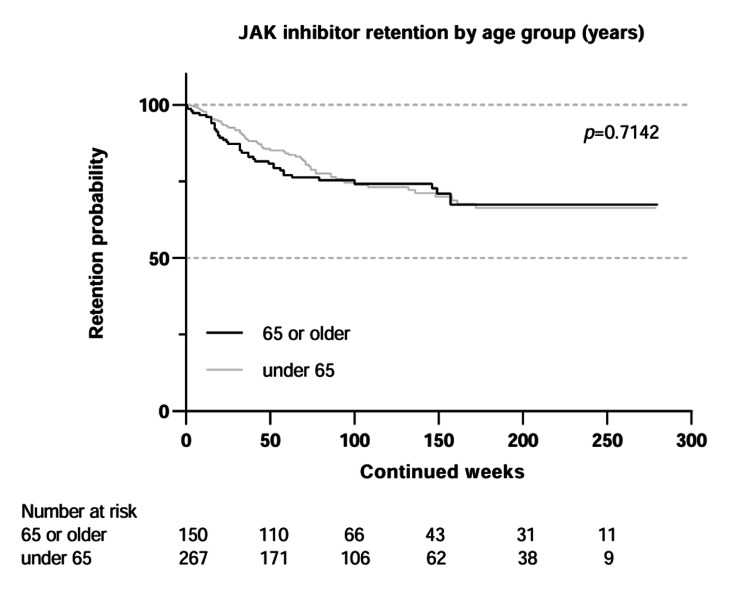
Kaplan–Meier survival curves comparing drug retention in patients aged <65 years versus ≥65 years Statistical comparison was performed using the log-rank test. JAK: Janus kinase

Kaplan-Meier curves were used to compare the drug retention of tofacitinib and baricitinib (Figure 2), upadacitinib and filgotinib (Figure 3), and patients under 65 years of age, and 65 years and older (Figure 4). 

Reason for discontinuation 

Reasons for discontinuation were classified into one of seven categories: primary inefficacy (34.3%), secondary inefficacy (5.9%), side effects (24.5%), non-toxic reasons (11.8%), recurrent infections (9.8%), MHRA alert (9.8%), and abnormal blood test results (3.9%). Demographic and disease characteristics for each of these reasons are shown in Table 4, including reasons for discontinuation for each drug.

**Table 4:** Patient characteristics by reason for JAK inhibitor discontinuation Values are presented as N (%) for categorical variables and mean ± SD for continuous variables. For ethnicity and drug rows, percentages are calculated within each row (i.e., across discontinuation reasons). For gender, diagnosis, and concomitant steroids, percentages are calculated within each column (i.e., within the reason subgroup). Units: ACPA (U/ml); RF (IU/ml). “–” indicates 0 or not applicable. JAK: Janus kinase; MHRA: Medicines and Healthcare products Regulatory Agency; RA: rheumatoid arthritis; ACPA: anti-citrullinated protein antibody; RF: rheumatoid factor; DAS-28 CRP: Disease Activity Score in 28 joints using C-reactive protein; csDMARDs: conventional synthetic disease-modifying antirheumatic drugs

Variable	Primary inefficacy (n=35)	Secondary inefficacy (n=6)	Side effects (n=25)	Non-toxic reasons (n=12)	Recurrent infections (n=10)	MHRA alert (n=10)	Abnormal blood results (n=4)	Total (n=102)
Ethnicity: White	29 (38.2%)	4 (5.3%)	19 (25.0%)	6 (7.9%)	8 (10.5%)	6 (7.9%)	4 (5.3%)	76 (74.5%)
Ethnicity: Asian	6 (26.1%)	2 (8.7%)	3 (13.0%)	6 (26.1%)	2 (8.7%)	4 (17.4%)	–	23 (22.5%)
Ethnicity: Mixed White & Black	–	–	3 (100.0%)	–	–	–	–	3 (2.9%)
Age (years)	58.4 ± 12.2	55.2 ± 9.0	59.0 ± 13.3	50.8 ± 19.9	59.1 ± 14.6	63.8 ± 12.0	59.5 ± 9.0	58.1 ± 13.6
Drug: Tofacitinib	11 (33.3%)	3 (9.1%)	3 (9.1%)	5 (15.2%)	1 (3.0%)	9 (27.3%)	1 (3.0%)	33 (32.4%)
Drug: Baricitinib	23 (40.4%)	3 (5.3%)	17 (29.8%)	4 (7.0%)	7 (12.3%)	–	3 (5.3%)	57 (55.9%)
Drug: Upadacitinib	1 (16.7%)	–	3 (50.0%)	1 (16.7%)	1 (16.7%)	–	–	6 (5.9%)
Drug: Filgotinib	–	–	2 (33.3%)	2 (33.3%)	1 (16.7%)	1 (16.7%)	–	6 (5.9%)
Gender: Female	27 (77.1%)	6 (100.0%)	23 (92.0%)	11 (91.7%)	9 (90.0%)	9 (90.0%)	4 (100.0%)	89 (87.3%)
Gender: Male	8 (22.9%)	–	2 (8.0%)	1 (8.3%)	1 (10.0%)	1 (10.0%)	–	13 (12.7%)
Diagnosis: Seropositive RA	23 (65.7%)	4 (66.7%)	16 (64.0%)	5 (41.7%)	6 (60.0%)	5 (50.0%)	4 (100.0%)	63 (61.8%)
Diagnosis: Seronegative RA	7 (20.0%)	1 (16.7%)	7 (28.0%)	5 (41.7%)	3 (30.0%)	3 (30.0%)	–	26 (25.5%)
Diagnosis: Psoriatic arthritis	5 (14.3%)	1 (16.7%)	2 (8.0%)	2 (16.7%)	1 (10.0%)	2 (20.0%)	–	13 (12.7%)
ACPA (U/ml)	185.3 ± 162.8	42.3 ± 53.9	118.6 ± 142.8	70.5 ± 103.2	202.7 ± 156.3	117.2 ± 138.0	305.3 ± 69.5	154.6 ± 149.0
RF (IU/ml)	211.7 ± 385.6	101.5 ± 119.4	123.6 ± 182.7	106.8 ± 110.4	49.8 ± 15.2	120.6 ± 204.7	143.0 ± 182.7	147.2 ± 262.6
DAS28-CRP (RA only)	5.5 ± 0.9	6.1 ± 0.8	5.5 ± 0.9	4.1 ± 1.0	6.2 ± 0.9	4.0	–	5.3 ± 1.0
Number of previous biologics (median ± IQR)	1 ± 1	1.5 ± 2.5	1 ± 1	1 ± 1.5	1 ± 1.75	1.5 ± 1	0 ± 0.25	1 ± 2
Number of concomitant csDMARDs (median ± IQR)	1 ± 0	0.5 ± 1.75	1 ± 1	1 ± 2	0 ± 0.75	1 ± 1.75	0.5 ± 1	1 ± 1
Concomitant steroids: Yes	9 (25.7%)	1 (16.7%)	8 (32.0%)	4 (33.3%)	3 (30.0%)	2 (20.0%)	1 (25.0%)	28 (27.5%)

For chi-square testing, reasons for drug discontinuation were categorised into three broader categories: inefficacy, adverse events, and others. We sought to determine whether there is any association between these outcomes and the patient baseline characteristics, and the results are shown in Table 5. 

**Table 5:** Associations between baseline characteristics and reason for JAK inhibitor discontinuation Statistical comparisons were performed using the chi-square (χ²) test of independence. Effect size is reported as Cramér’s V (equivalent to φ for 2×2 tables). Degrees of freedom (df) are calculated as (rows−1)×(columns). Outcome categories were grouped as: inefficacy, adverse events, and other. † Ethnicity analysis excluded Mixed White & Black patients due to low counts (n=3); ‡ Drug analysis compared baricitinib vs tofacitinib; upadacitinib and filgotinib were excluded due to small numbers (n=6 each); § DAS28-CRP analysis included RA patients. JAK: Janus kinase; RA: rheumatoid arthritis; DAS-28 CRP: Disease Activity Score in 28 joints using C-reactive protein; csDMARDs: conventional synthetic disease-modifying antirheumatic drugs

Variable	χ² value	df	p value	n (used)	Effect size (Cramér’s V)
Ethnicity†	8.902	2	0.012	99	0.300
Age (<65 vs ≥65 years)	2.294	2	0.318	102	0.150
Gender	2.568	2	0.277	102	0.159
Drug (baricitinib vs tofacitinib)‡	18.843	2	<0.001	90	0.458
Diagnosis	4.215	4	0.378	102	0.144
No previous biologics (0,1,2,≥3)	5.340	6	0.501	102	0.162
Concomitant csDMARDs (0,1,≥2)	10.004	4	0.040	102	0.221
Concomitant glucocorticoid (yes/no)	0.605	2	0.739	102	0.077
DAS28-CRP (moderate vs high)§	13.448	2	0.001	29	0.681

Significance (p<0.05) was present for ethnicity, drug, DAS28-CRP (only RA patients), and number of concomitant csDMARDs. The results for these characteristics are shown in Table 6. For ethnicity, there were only three patients identified as mixed white and black, and due to low numbers, these patients were excluded (all discontinued their JAK inhibitor due to side effects). The majority of patients were White or Asian. For the drug, tofacitinib was compared against baricitinib. Upadacitinib and filgotinib were excluded due to low numbers (only six cases each). DAS28-CRP values (before commencing the JAK inhibitor) were available in 29 RA patients and were grouped as moderate or severe disease activity. 

**Table 6:** Distribution of discontinuation reasons by ethnicity, JAK inhibitor, concomitant csDMARDs, and DAS28-CRP at commencement Values are N (% within subgroup). Statistical comparisons used the chi-square (χ²) test of independence. Inefficacy = primary + secondary inefficacy; adverse event = side effects + recurrent infection + abnormal blood results; other = non-toxic reasons + MHRA alert. Drug χ² and p-values are for baricitinib vs tofacitinib (upadacitinib and filgotinib excluded due to low n); ethnicity χ² and p-value exclude the Mixed group due to low counts (n=3). DAS28-CRP analysis includes RA patients with baseline DAS28 and compares moderate vs high at commencement. JAK: Janus kinase; MHRA: Medicines and Healthcare products Regulatory Agency; RA: rheumatoid arthritis; χ²: chi-square; DAS-28 CRP: Disease Activity Score in 28 joints using C-reactive protein; csDMARDs: conventional synthetic disease-modifying antirheumatic drugs

Group	Subgroup (n)	Inefficacy	Adverse event	Other	Test statistic (χ²)	p value
Ethnicity	White (n=76)	33 (43.4%)	31 (40.8%)	12 (15.8%)	8.902	0.012
	Asian (n=23)	8 (34.8%)	5 (21.7%)	10 (43.5%)		
Drug	Tofacitinib (n=33)	14 (42.4%)	5 (15.2%)	14 (42.4%)	18.843	<0.001
	Baricitinib (n=57)	26 (45.6%)	27 (47.4%)	4 (7.0%)		
Number of concomitant csDMARDs	0 (n=34)	9 (26.5%)	18 (52.9%)	7 (20.6%)	10.004	0.040
	1 (n=46)	24 (52.2%)	15 (32.6%)	7 (15.2%)		
	≥2 (n=22)	10 (45.5%)	5 (22.7%)	7 (31.8%)		
DAS28-CRP at commencement (RA only)	Moderate (n=9)	2 (22.2%)	2 (22.2%)	5 (55.6%)	13.448	0.001202
	High (n=20)	9 (45.0%)	11 (55.0%)	0 (0.0%)		

For age, in addition to chi-square comparing those under 65 and those 65 and older, a Kruskal-Wallis H test showed that there was no statistically significant difference (p=0.633) in age between the three groups who discontinued due to inefficacy, adverse events, and other reasons. 

## Discussion

This study provides real-world data on JAK inhibitor discontinuation rates and reasons for discontinuation in a multi-ethnic cohort in a UK tertiary centre. 

Discontinuation and retention rates 

Tofacitinib had the highest proportion of discontinuations at 51.6%, followed by baricitinib, 23.5%; filgotinib, 12.2%; and upadacitinib, 9.8%. Previous studies have also reported different discontinuation rates for the JAK inhibitors [15]. In our study, baricitinib had a shorter mean time to discontinuation at 9.9 months when compared to tofacitinib at 16.1 months. Differences in patient baseline characteristics were seen between these two groups, with a higher proportion of the patients who discontinued baricitinib having seropositive RA and being on concomitant glucocorticoids, and a subset of the tofacitinib patients having PsA. Upadacitinib and filgotinib were associated with high early discontinuation rates, although the small sample size precludes definitive conclusions.

Reasons for discontinuation 

Primary inefficacy (34.3%) was the most common reason for JAK inhibitor discontinuation in our study, followed by side effects (24.5%). Interestingly, previous studies have reported that JAK inhibitors are more often discontinued due to adverse events than inefficacy [15-17]. The European Medicines Agency (EMA) defines an adverse event as “an untoward medical occurrence after exposure to a medication, which is not necessarily caused by that medicine [18].” In our study, discontinuations due to adverse events were 38.2% (including side effects, recurrent infections, and abnormal blood results), while inefficacy (both primary and secondary) was 40.2%. 

This difference is possibly due to the characteristics of the patient populations, including the degree of comorbidities and concomitant medications. These factors could affect the proportion of discontinuations due to adverse events versus inefficacy. In our study, primary inefficacy was the primary cause of baricitinib and tofacitinib discontinuation, whereas side events were among the most common reasons for upadacitinib and filgotinib. 

Safety concerns and regulatory influence 

JAK inhibitor safety has recently been a subject of increasing study, particularly following ORAL Surveillance [7], which demonstrated a higher incidence of cardiovascular events and malignancies with tofacitinib compared to TNF inhibitors, leading to regulatory warnings from the MHRA and Food and Drug Administration. EULAR recommended that JAK inhibitors be used after careful consideration of risk factors, including age 65 years or older, smoking history, and risk factors for cardiovascular disease, malignancy, and thromboembolic events [8]. These concerns were reflected in our data, where discontinuations due to MHRA alerts were recorded in 9.8% of cases. Tofacitinib and filgotinib were the only JAK inhibitors discontinued for this reason, and the patients in this category had the highest mean age at 63.8 years.

Impact of baseline characteristics on the reason for discontinuation 

We found significant associations between ethnicity, the specific JAK inhibitor used, the number of concomitant csDMARDs, and DAS28-CRP in the RA patients with discontinuation reasons. Significance was not seen for age group (<65 vs ≥65 years), gender, diagnosis, number of previous biologics, and concomitant steroids.

Ethnicity influenced treatment discontinuation patterns, with a higher proportion of adverse event-related discontinuations in White patients than Asian patients (p=0.012). This aligns with prior studies suggesting ethnic variations in treatment tolerability, potentially driven by pharmacogenomic differences [19,20]. However, evidence on ethnic differences in JAK-inhibitor response is limited; in a UK single-centre cohort of baricitinib-treated RA, DAS28 responses were similar in Asian and White patients [21].

The proportion of adverse event-related discontinuations was higher in the baricitinib group than in the tofacitinib group (p<0.001), possibly reflecting differences in safety profiles. The proportion of inefficacy-related discontinuations was higher in patients on one, two, or more concomitant csDMARDs, whereas the proportion of adverse event-related discontinuations was higher in those on JAK inhibitor monotherapy (p=0.040). This may reflect greater attribution of adverse events in monotherapy compared to combination therapy. Furthermore, a higher number of concomitant DMARD uses may indirectly indicate a difficult-to-control disease. Among patients with a baseline DAS28-CRP, those with high disease activity were more likely to discontinue their JAK inhibitor for inefficacy or adverse events, whereas those with moderate disease activity more often stopped for other reasons.

Limitations 

This study has the following limitations. Firstly, the reasons for JAK inhibitor discontinuation were recorded from clinician documentation, which may not fully capture patient-reported concerns or multifactorial reasons for discontinuation. Furthermore, clinical information was not uniformly available for all patients, owing to the retrospective study design. 

Additional limitations include the following: The sample sizes for upadacitinib and filgotinib were small, limiting the conclusions that can be made about these agents, and long-term safety outcomes were not assessed, which is important given the safety concerns with JAK inhibitors. It is also possible that, given the growing safety concerns with JAK inhibitors, and especially tofacitinib, clinicians may be highly selective and preferentially prescribe certain JAK inhibitors to patients at lower risk of adverse events. This prescribing behaviour could mean fewer discontinuations due to adverse events and change overall discontinuation patterns.

## Conclusions

This study provides real-world insights into JAK inhibitor discontinuation reasons in a UK tertiary centre cohort. Primary inefficacy was the most common reason for discontinuation for all JAK inhibitors, and there were differences in discontinuation patterns among the different drugs. This suggests that drug-specific factors may affect retention. Future studies should explore the impact of patient baseline characteristics on drug safety and efficacy to help optimise treatment decisions and also determine whether discontinuation patterns reflect a class-wide effect of JAK inhibitors or if individual drugs have distinct effects. 
